# Research Coordinators’ Perspectives on Recruitment of Minoritized People with Cystic Fibrosis into Clinical Trials

**DOI:** 10.21203/rs.3.rs-5195002/v1

**Published:** 2024-11-13

**Authors:** Tijana Milinic, Nora Burdis, Eliana Gill, Patricia Burks, Mary Elizabeth Jarosz, Amy J. Hoffman, Siddhartha G. Kapnadak, Kathleen J. Ramos, Christopher H. Goss

**Affiliations:** University of Washington; University of Washington; University of Washington; Cystic Fibrosis Foundation; Cystic Fibrosis Foundation; Seattle Children’s Research Institute; University of Washington; University of Washington; University of Washington

**Keywords:** equity, cystic fibrosis, clinical trial recruitment, disparities

## Abstract

**Background:**

Clinical trials in cystic fibrosis (CF) disproportionately over-represent non-Hispanic, White individuals. Barriers and facilitators to enrolling racially and ethnically minoritized individuals with CF are not well understood. This study explored research coordinator (RC) perspectives on recruitment and enrollment of minoritized people with CF (PwCF) into clinical trials.

**Methods:**

A cross-sectional survey was administered to RCs in the CF Therapeutics Development Network (CF TDN), eliciting perceived barriers and facilitators to inclusion of minoritized PwCF in clinical research. Respondents were categorized based on their self-reported experience discussing and successfully enrolling minoritized PwCF into clinical trials.

**Results:**

Among 48 respondents, the majority (n = 31, 64%) had little to no experience discussing CF clinical trials with minoritized PwCF. Respondents who had a moderate or great deal of experience identified that having a trusted clinical team member first introduce the study to PwCF as the most common strategy for recruitment. Experienced respondents also identified the importance of having team members who speak the same language or are the same culture as the minoritized PwCF. Respondents who had little to no experience successfully enrolling minoritized PwCF into clinical trials cited low numbers of minoritized patients at their study center as an important consideration. All respondents emphasized language barriers in enrollment including need for adequate translation services and printed materials in the PwCF’s primary language.

**Conclusion:**

Our study identified modifiable barriers that may be addressed at the level of trial design and study center.

## Background

In 2022, 8.8% of people with CF (PwCF) in the Cystic Fibrosis Foundation (CFF) Patient Registry Annual Report identified as non-White and 10% as Hispanic [1]. Black and Hispanic individuals make up a growing number of individuals with CF, and it is well established that these patients experience higher morbidity and mortality as compared to non-Hispanic White patients [2–6]. Inequity in access to care and social determinants of health have resulted in poorer nutritional status, as measured by height and weight percentiles for Black individuals with CF, as well as reduced lung function, as measured by forced expiratory volume in one second (FEV_1_), even after controlling for genotype [2]. Hispanic patients are at an increased risk of acquiring pulmonary infections at an earlier age, and are at three-times increased risk of mortality compared to non-Hispanic CF patients [3–6]. Clinical research that disproportionately over includes non-Hispanic White PwCF widens these existing disparities [7, 8]. Limited literature outside of CF identifies many barriers to research participation including time burden, transportation, distance to study centers, and financial limitations [9–12]. Study communication and teaching that does not appropriately address unique language and cultural practices, particularly in regards to experimental treatment arms and informed consent, further excludes minoritized groups from clinical studies [11, 13, 14]. Research coordinators (RCs) play a key role in recruiting participants for clinical trials. Although cultural competency, community engagement/involvement, and diversity of research teams are important in enrolling a diverse population [15, 16], there are no studies evaluating RCs’ perspectives on this subject in CF. Inclusion that is representative of the whole CF population is vital to understanding adverse drug events and therapeutic effects. Further, as we search for treatments for modulator-ineligible patients, Black and Hispanic PwCF are critical to enroll in trials as they are more likely to be ineligible for CF transmembrane conductance regulator (CFTR) modulator therapy due to genotype [17]. This study aimed to understand RCs’ perspectives of barriers and facilitators to the recruitment of diverse populations into CF clinical research.

## Methods

### Study Design and Participants:

A cross-sectional survey was designed based on prior research evaluating barriers and facilitators to recruitment of diverse populations in clinical research [7–13]. Survey questions addressed years of RC experience, the extent of experience discussing clinical trials with minoritized people with CF, the extent of experience successfully enrolling minoritized PwCF to clinical trials, and perceptions of minoritized PwCF’s barriers and facilitators to enrollment in clinical trials (see Supplement). Discussion of clinical trials and factors facilitating or inhibiting discussion were explored through multiple choice survey items. Enrollment into clinical trials and perceptions of barriers and facilitators to successful enrollment of minoritized PwCF was explored through open ended response questions. In this survey, minoritized individuals were defined as people who identify with Hispanic ethnicity and/or non-White race. Between April 27 2021 to May 14, 2021, the anonymized survey was distributed to RCs in the CF TDN via a link in the TDN Digest (emailed newsletter). The TDN Digest is distributed nationally to primary investigators, study staff, and all research coordinators within the CF TDN. Only RCs were invited to participate in the survey. The University of Washington IRB approved this study (Study #00018252). The IRB provided a waiver of documentation of informed consent since the study was determined to be minimal in risk.

### Data Analysis:

Data were entered into Microsoft Excel (Microsoft, Redmond, WA, USA) spreadsheet for analysis. Survey responses were summarized using descriptive statistics. Qualitative analysis was performed for all survey free-text response answers using deductive analysis. Investigators initially read through all free text answers, noting emergent themes. Then three coders separately coded all responses. After coding, investigators met to review codes and resolve discrepancies (AJH, PB, MEJ). A final codebook was generated and the frequency of each code was recorded.

## Results

The survey was distributed to the CF TDN listserv, with a total distribution list including RCs at 91 CF centers. We received a total of 53 anonymous survey responses. Of the 53 survey respondents, 42% had greater than 10 years of CF RC experience.

### Discussing Clinical Trials

**Figure 1:**
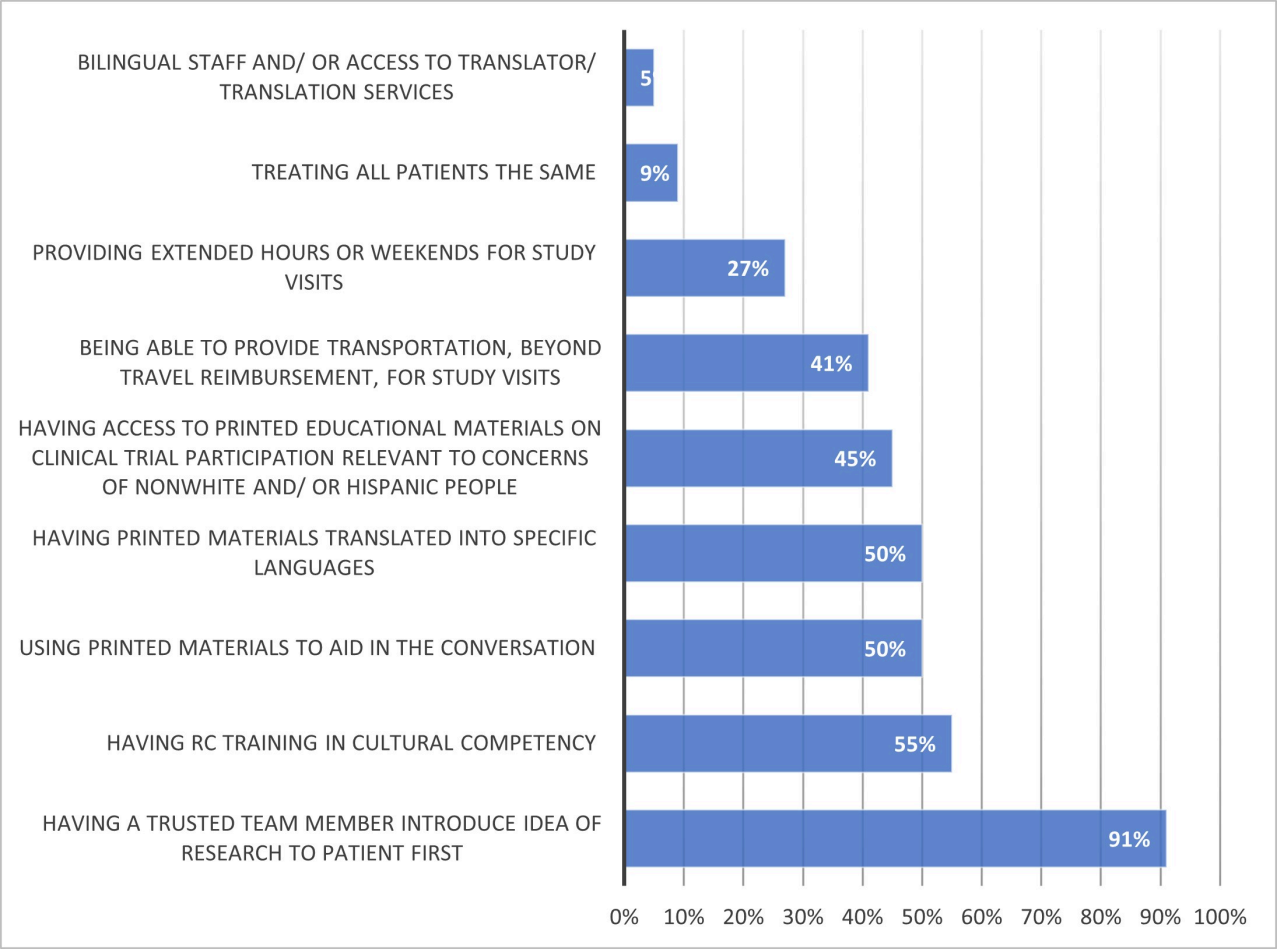
Which of the following have you found helpful when discussing a specific CF clinical trial with nonwhite and/or Hispanic people? (Check all that apply) Among respondents with great deal of experience.

In total 49 RCs reported their level of experience discussing clinical research with diverse populations. Of these, only 22 (45%) reported a moderate or great deal of experience discussing clinical trials with minoritized PwCF. Of these 22 respondents with moderate or great deal of experience (Fig. 1), 20 (91%) cited that the most common strategy that facilitates discussion of clinical trials is having a trusted clinical team member first introduce the idea of research to PwCF. Other useful tools cited by RCs with experience discussing clinical trials included having RC training in cultural competency (55%), using printed materials translated into specific languages (50%), having access to printed educational material on clinical trial participation relevant to the concerns of minoritized PwCF (45%), and being able to provide transportation beyond travel reimbursement for study visits (41%). Respondents with little or no experience (n = 25) discussing clinical trials cited low numbers of people of color and/or Hispanic PwCF as major reasons for their inexperience in discussing clinical research with minoritized patients.

### Successfully Enrolling into Clinical Trials

Of 48 RCs who reported their experience enrolling diverse populations, the majority (n = 31, 64%) reported little to no experience successfully enrolling minoritized PwCF into clinical trials specifically. Barriers and facilitators to enrolling diverse populations were explored through open ended responses.

**Figure 2:**
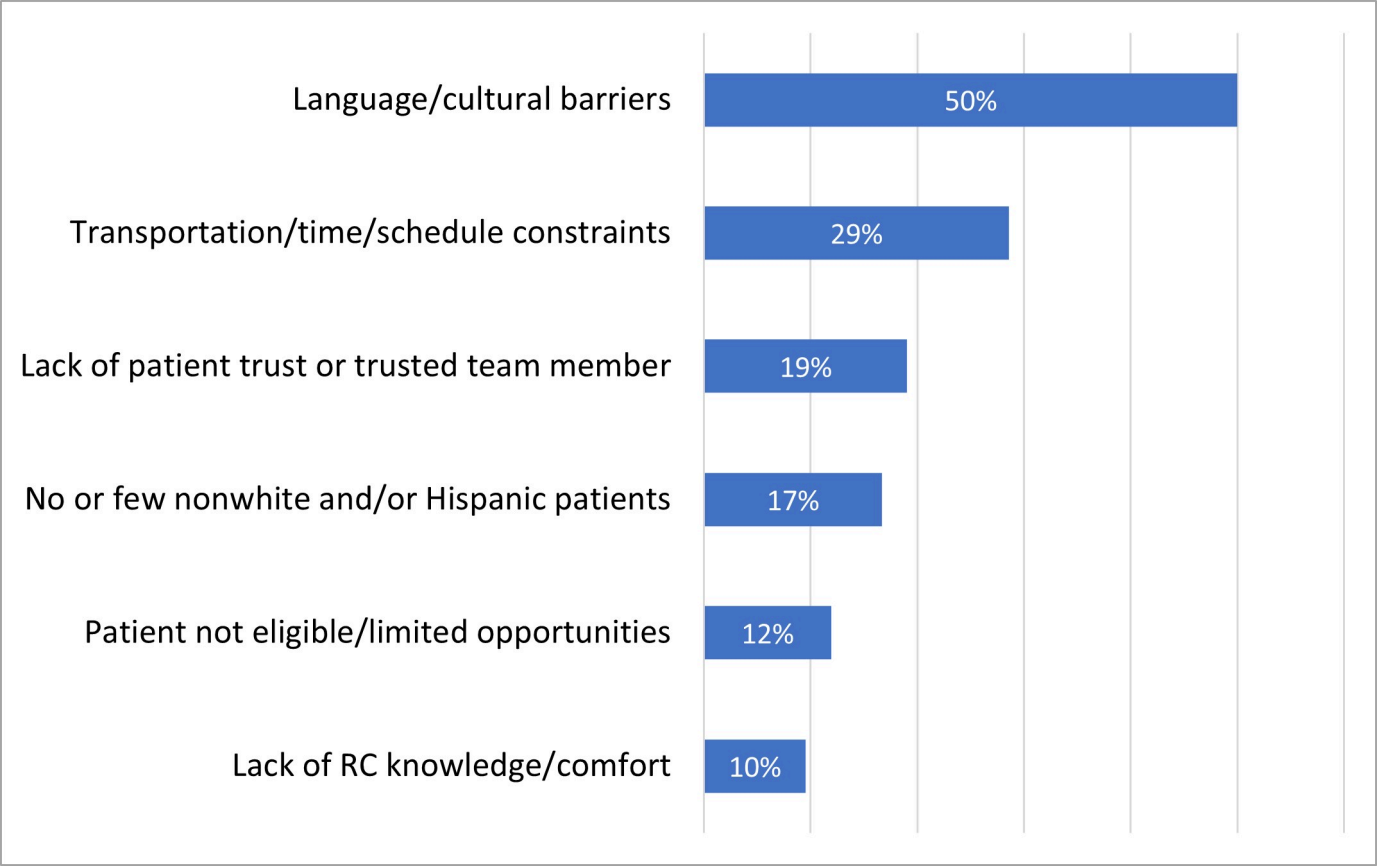
Barriers to enrolling minoritized people with CF in CF clinical trials (open ended responses), among respondents with all levels of experience

Of (n = 31) respondents who reported little to no experience successfully enrolling non-white and Hispanic individuals into CF clinical trials, 47% cited that there are few non-White and/ or Hispanic individuals at their centers or few who are eligible for studies in open ended responses. Among RCs with moderate to a great deal of experience successfully enrolling minoritized patients into clinical trials (n = 17), a smaller fraction of respondents (24%) cite that their centers have small/ limited populations. Across all levels of experience with diverse recruitment (n = 42), language and cultural practices were the most common barriers to enrolling non-white and/ or Hispanic people in CF clinical trials. Other commonly cited barriers among all respondents included transportation/time/schedule constraints involved in study participation and lack of patient trust (Fig. 2).

**Figure 3:**
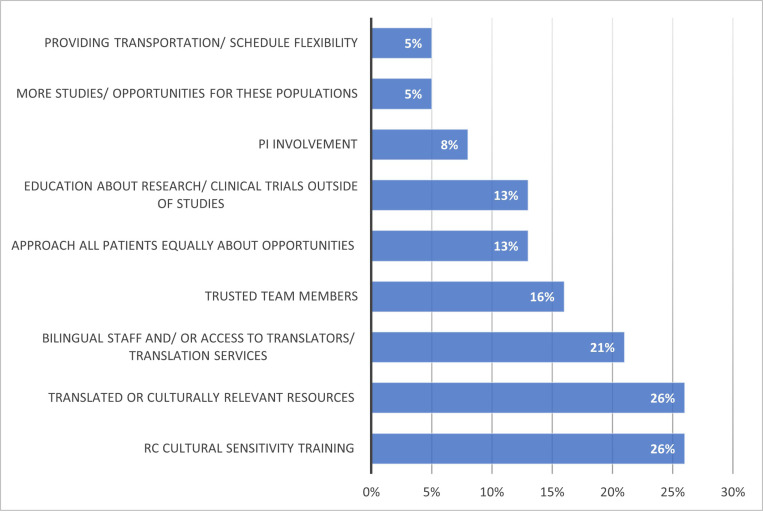
What do you think would help teams be more effective in enrolling minoritized people with CF? (open ended response) Among respondents with all levels of experience

Among those who responded (n = 38), the most frequently reported strategies (Fig. 3) to improve diversity of enrollment into clinical trials included RC cultural sensitivity training (cited by 26%), translated or culturally relevant resources (26%), and bilingual staff and/ or access to translation services (21%). When asked why they have been successful in enrolling diverse PwCF into CF clinical trials, RCs with moderate to significant experience cite that having a team member who speaks the language or who is from the same culture facilitates inclusion (Fig. 3). Other common strategies were treating and approaching all PwCF the same, taking time to explain the study and provide relevant resources, as well as time to connect and build relationships with the PwCF.

## Discussion

Our study identified key RC perspectives on the recruitment and enrollment of diverse populations of PwCF in clinical research. We found that many RCs had little to no experience discussing and enrolling minoritized PwCF into clinical trials. An important strategy to help facilitate discussion of clinical trials with minoritized PwCF was to first allow a trusted clinical team member to introduce clinical trial participation. Similar to prior research outside of CF, important modifiable barriers included language and cultural practices, as well as transportation, time, and schedule constraints. Recent research outside of the CF population confirms that lack of funding for translation services, especially in non-industry sponsored trials, limits enrollment of non-English speaking patients [18]. In our study, we found that language barriers appear to be very important, and reliable translation services can be lacking despite the resources available among this well-funded network.

Importantly, small numbers of minoritized PwCF were also identified as a barrier among respondents. On one hand, this may simply reflect a relative a lack of individuals available for study recruitment. However, particularly with growth of the non-white CF population [1] and the previously demonstrated under-representation of this subset in CF clinical trials [7–8], it may be more productive to consider lower numbers as a factor that hinders proper training and preparation that would otherwise allow RCs and/or providers to more effectively discuss trial enrollment with minoritized PwCF. The fact that RCs with little to no experience with diverse populations in our study were nearly twice as likely to report small numbers as a barrier to recruitment compared to those with moderate to a great deal of experience (47% vs. 24%, respectively) perhaps supports this point and argues for strengthening of culturally sensitive training in the CF research community. Other important strategies may include hiring team members who speak the same language or share the culture of PwCF at the study center.

Black and Hispanic people with CF not only experience a greater burden of disease but prior research has shown delayed CF diagnosis for these communities [19, 20]. To compound these disparities, Black and Hispanic individuals with CF are more likely to have CFTR mutations that are ineligible for life saving modulator therapy [17]. Future clinical trials will focus on this subset of patients as we search for targeted therapies for rare mutations. It is imperative that focus is shifted to understand how to improve engagement among minoritized communities. Current recruitment practices have not been successful at enrolling populations that fully represent the CF community. Deficit based approaches that place the burden on patients and their families are not appropriate to address the issue. Interventions at the site and study level, as our study describes, will be necessary in addition to large scale cultural and societal level shifts as well as programmatic changes to help overcome logistical barriers to participation.

Our study had several limitations. Since the study did not include respondent demographics, we were unable to evaluate the demographic impact on perceived comfort and experience with enrolling minoritized PwCF. Additionally, we are not able to determine the exact response rate across the network due to variability in the number of RCs at CF TDN centers and a lack of reporting of CF TDN sites in the survey.

### Conclusion

Our study confirms prior findings on the importance of cultural sensitivity training and uniquely highlights the importance of hiring diverse research teams to improve experience in the recruitment of minoritized PwCF in clinical trials [15, 16]. Further research is needed to better understand the differences in clinical trial enrollment success at centers with higher proportions of minoritized PwCF compared to those with fewer. Qualitative interviews of RCs in the CF TDN may help further describe useful recruitment strategies and characterize barriers to equitable enrollment. This study highlights possible intervenable barriers and facilitators to equitable and representative enrollment in CF clinical trials, an area of significantly growing importance as we search for novel therapies.

## Supplementary Material

Supplement 1

## Data Availability

The datasets used and/ or analyzed during the current study are available from the corresponding author on reasonable request.

## Abbreviations

CF: cystic fibrosis
RCs: research coordinators
CFTR: CF transmembrane conductance regulator
PwCF: persons/people with cystic fibrosis
CF TDN: CF Therapeutics Development Network
