# Supplementary material for: Research Coordinators’ Perspectives on Recruitment of Minoritized People with Cystic Fibrosis into Clinical Trials
Source: Res Sq. 2024 Nov 13:rs.3.rs-5195002. Preprint. [Version 1] doi: 10.21203/rs.3.rs-5195002/v1 (PMC11601850; doi:10.21203/rs.3.rs-5195002/v1)
Supplement: Supplement 1 [file NIHPPRS5195002V1-supplement-1.pdf]

Supplementary Files

This is a list of supplementary files associated with this preprint. Click to download.

- CFClinicalTrialRecruitmentManuscriptSupplement.docx
